# Trigger Wrist Caused by a Rheumatoid Nodule on the Flexor Pollicis Longus Tendon

**DOI:** 10.31486/toj.22.0033

**Published:** 2023

**Authors:** Devan Higginbotham, Dominik Fleifel, Andrew Tsai

**Affiliations:** Department of Orthopedic Surgery, Detroit Medical Center, Detroit MI

**Keywords:** *Arthritis–rheumatoid*, *hand*, *trigger finger disorder*, *trigger wrist*

## Abstract

**Background:** Trigger wrist is a rare condition. Previously reported cases have involved nodules or ganglion cysts affecting flexor digitorum profundus tendons; however, we found no reported cases of trigger wrist caused by a rheumatoid nodule on the flexor pollicis longus tendon.

**Case Report:** A 57-year-old female presented with the complaint of chronic triggering of the right thumb and numbness in her fingers consistent with carpal tunnel syndrome. Corticosteroid injection did not provide symptom relief, so the patient was scheduled for surgery. A 3 × 1.5-cm lesion was removed from the flexor pollicis longus tendon distal to the carpal tunnel. Histopathologic examination demonstrated that the lesion was a rheumatoid nodule.

**Conclusion:** Patients with rheumatoid arthritis who present with trigger finger symptoms of the thumb with concomitant carpal tunnel symptoms require careful evaluation to rule out trigger wrist before the condition progresses to Mannerfelt lesion.

## INTRODUCTION

Trigger finger is one of the most common disorders of the hand.^1^ Compared to trigger finger, trigger wrist is a relatively rare condition, and triggering of the fingers secondary to a lesion at the wrist is an uncommon phenomenon. Multiple causes of trigger wrist have been described, with lesions reported as lipomas, fibromas, anomalous skeletal muscles, tophi, synovitis of the carpal tunnel, pigmented villonodular synovitis, and rheumatoid nodules.^2-9^ Most commonly, trigger wrist has been described as occurring on the flexor tendons.^10^ To our knowledge, trigger wrist caused by a rheumatoid nodule on the flexor pollicis longus tendon has yet to be described.

Rheumatoid nodules of the upper extremity are relatively common in patients with rheumatoid arthritis, seen in up to 25% of those patients.^11^ Research suggests that the etiology of rheumatoid nodules is related to immune-complex-mediated small vessel vasculitis in areas of high use or trauma.^11^ These lesions can lead to pain, nerve compression, mechanical block from tendon or joint involvement, and infection.^12^

## CASE REPORT

**Figure 1.**
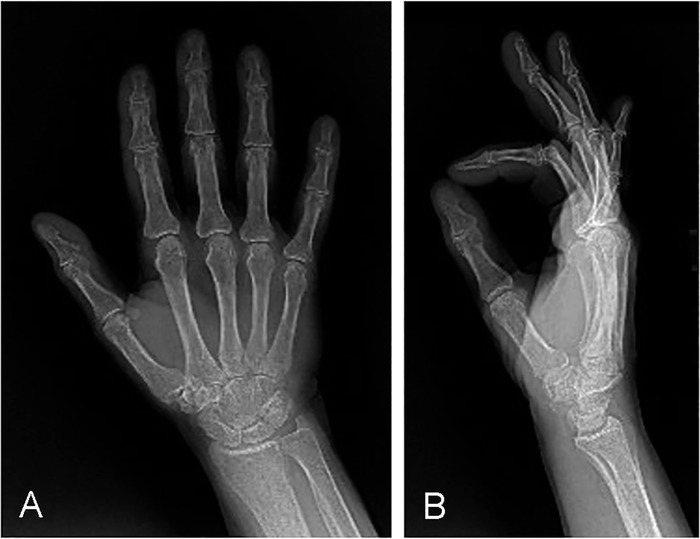
(A) Posteroanterior and (B) lateral radiographs show mild degenerative joint disease of the interphalangeal and carpometacarpal joints of the thumb.

A 57-year-old right-hand-dominant female with history of rheumatoid arthritis presented to the clinic with complaints consistent with carpal tunnel syndrome of the right wrist, synovitis of the flexor tendons, trigger thumb that did not present as a normal trigger thumb, and pain in her hand and wrist. On examination, the patient had noticeable swelling and persistent thumb triggering with numbness in her index, middle, and ring fingers. The triggering of the thumb appeared to be coming from the carpal tunnel because no palpable lesion surrounded the A1 pulley as seen in a typical trigger thumb. Radiographs of the right thumb revealed mild osteoarthritic changes over the interphalangeal joint (Figure 1) but no scaphoid-trapezium-trapezoid joint degenerative changes. Electrodiagnostic studies demonstrated severe median neuropathy on the right with evidence of ongoing denervation of the right abductor pollicis brevis muscle.

The patient received a corticosteroid injection for her right trigger thumb over the A1 pulley, but the injection did not provide any symptom relief. The working diagnosis was locked trigger thumb or wrist, and the patient was scheduled for surgical release of the cause of the triggering, carpal tunnel release, and excision of synovitis around the flexor tendons caused by swelling and poorly controlled rheumatoid arthritis.

The carpal tunnel release was performed, and the median nerve was noted to be extremely compressed, assuming an hourglass-type shape underneath the carpal tunnel. Inflamed synovium covered all the tendons, both the deep and superficial flexor tendons. Attempts were made to remove all of the synovium. The thumb tendons were explored. Intraoperatively, the patient was asked to flex and extend her thumb, with no further triggering of the digit. Exploration of the flexor pollicis longus tendon demonstrated a 3 × 1.5-cm lesion affixed to the flexor pollicis longus tendon just distal to the carpal tunnel. The lesion did not appear to be a ganglion cyst or giant cell tumor. The lesion was bluntly dissected, excised, and sent to pathology.

Further examination of the flexor pollicis longus tendon demonstrated a large area of tendon disruption along the lesion that measured approximately 3 to 4 cm in length and encompassed approximately one-third to one-half of the tendon. The tendon was much thinner in this region compared to the areas proximal and distal to the lesion. The lesion was concerning for an early Mannerfelt lesion because of repetitive rubbing of the tendon on the lesion. The base of the carpal tunnel was palpated, and no protruding osteophytes or other obvious abnormality could be found in the carpal tunnel or carpometacarpal region to explain her lesion. The tendon was repaired directly with a running 5-0 dissolvable suture to minimize any stray fragments.

**Figure 2.**
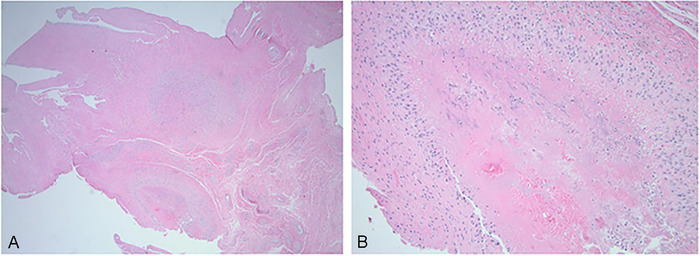
Rheumatoid nodule removed from the flexor pollicis longus tendon has a central area of necrotic tissue with a thick border of fibroblasts ([A] magnification × 2 and [B] magnification × 10).

Pathology report described the lesion as a rheumatoid nodule with reactive hyaline changes and synovial tissue with hyperplastic changes (Figure 2).

At 3-month follow-up, the patient reported improvements with sensation in the median nerve distribution of her hand, complete resolution of the mechanical locking symptoms of her thumb, and no postoperative complications.

## DISCUSSION

Marti first described trigger wrist in 1960,^13^ and in 2016, Park et al suggested the following 3 cardinal symptoms: (1) finger triggering at the wrist during finger motion, often with more than 2 digits involved; (2) mild to moderate paresthesia of the hand presenting as carpal tunnel syndrome; and (3) crepitus with a swelling or palpable moving mass over the wrist.^9^ Arumugam et al described evaluating for an absence of tenderness over the A1 pulley in patients with trigger finger to suggest possible trigger wrist.^14^

As case reports of trigger wrist have been published, attempts have been made to create a classification system. In 1985, Suematsu et al reported on three types of triggering of the wrist: (1) type A, a mass occurring on the flexor tendon or flexor tendon sheath as it enters and leaves the carpal tunnel; (2) type B, an anomalous muscle belly entering and leaving the carpal tunnel (including an abnormal lumbrical muscle or abnormal muscle belly of the flexor digitorum superficialis); and (3) type C, a combination of tumor and anomalous muscle.^10^ The lesion causing trigger wrist in our patient was located on the flexor pollicis longus tendon. While Suematsu et al provided a good overall classification of trigger wrist types, more recent case reports demonstrate the need for an updated system, as prior classification systems do not contain comprehensive etiologies.^2^

In our patient, daily use of the flexor pollicis longus with the rheumatoid nodule in the carpal tunnel induced hypertrophic tenosynovitis. Similar mechanisms have been described with hypertrophied lumbrical muscles.^15^ Rheumatoid nodules located on extensor carpi radialis longus and flexor digitorum superficialis tendons have been documented to cause trigger wrist.^5^ Additionally, Giannikas et al described a case in which the combination of a rheumatoid nodule on the flexor digitorum profundus tendon and extensive synovitis caused ruptures of the superficial flexor tendons and trigger wrist.^16^ If allowed to progress, our patient's lesion likely would have resulted in a Mannerfelt lesion, which occurs when the flexor pollicis longus tendon ruptures from wear against an osteophyte in the carpal tunnel. These lesions are more common in patients with rheumatoid arthritis, and the flexor pollicis longus is the most widely known flexor tendon to rupture.^17^ The scaphoid was evaluated intraoperatively in our patient and was not noted to contain osteophytes. Given time for her rheumatoid arthritis to progress, the patient likely could have developed a Mannerfelt lesion because of tendon irritation from the rheumatoid nodule.

## CONCLUSION

While rheumatoid nodules located on the extensor carpi radialis longus, flexor digitorum superficialis, and flexor digitorum profundus tendon have been documented to cause trigger wrist, a rheumatoid nodule located on the flexor pollicis longus tendon causing trigger wrist is an uncommon entity that to our knowledge has not been reported in the literature. In patients with rheumatoid arthritis who present with trigger finger locking symptoms of the thumb with concomitant carpal tunnel symptoms, clinicians must evaluate for possible rheumatoid nodules on the flexor pollicis longus tendon.

## Notes

*This article meets the Accreditation Council for Graduate Medical Education and the American Board of Medical Specialties Maintenance of Certification competencies for Patient Care and Medical Knowledge.*
